# Nomogram incorporating preoperative pan-immune-inflammation value and monocyte to high-density lipoprotein ratio for survival prediction in patients with colorectal cancer: a retrospective study

**DOI:** 10.1186/s12885-024-12509-x

**Published:** 2024-06-17

**Authors:** Qinghua Liu, Haohao Wang, Qingjie Chen, Ruiying Luo, Changjiang Luo

**Affiliations:** Department of General Surgery, The Second Hospital of Lanzhou University, Lanzhou, China

**Keywords:** Colorectal cancer, Pan-immune-inflammation value, Monocyte to high-density lipoprotein ratio, Prognosis, Nomogram, Overall survival

## Abstract

**Objective:**

Using the preoperative pan-immune-inflammation value (PIV) and the monocyte to high-density lipoprotein ratio (MHR) to reflect inflammation, immunity, and cholesterol metabolism, we aim to develop and visualize a novel nomogram model for predicting the survival outcomes in patients with colorectal cancer (CRC).

**Methods:**

A total of 172 patients with CRC who underwent radical resection were retrospectively analyzed. Survival analysis was conducted after patients were grouped according to the optimal cut-off values of PIV and MHR. Univariate and multivariate analyses were performed using Cox proportional hazards regression to screen the independent prognostic factors. Based on these factors, a nomogram was constructed and validated.

**Results:**

The PIV was significantly associated with tumor location (*P* < 0.001), tumor maximum diameter (*P* = 0.008), and T stage (*P* = 0.019). The MHR was closely related to gender (*P* = 0.016), tumor maximum diameter (*P* = 0.002), and T stage (*P* = 0.038). Multivariate analysis results showed that PIV (Hazard Ratio (HR) = 2.476, 95% Confidence Interval (CI) = 1.410–4.348, *P* = 0.002), MHR (HR = 3.803, 95%CI = 1.609–8.989, *P* = 0.002), CEA (HR = 1.977, 95%CI = 1.121–3.485, *P* = 0.019), and TNM stage (HR = 1.759, 95%CI = 1.010–3.063, *P* = 0.046) were independent prognostic indicators for overall survival (OS). A nomogram incorporating these variables was developed, demonstrating robust predictive accuracy for OS. The area under the curve (AUC) values of the predictive model for 1-, 2-, and 3- year are 0.791,0.768,0.811, respectively. The calibration curves for the probability of survival at 1-, 2-, and 3- year presented a high degree of credibility. Furthermore, Decision curve analysis (DCA) for the probability of survival at 1-, 2-, and 3- year demonstrate the significant clinical utility in predicting survival outcomes.

**Conclusion:**

Preoperative PIV and MHR are independent risk factors for CRC prognosis. The novel developed nomogram demonstrates a robust predictive ability, offering substantial utility in facilitating the clinical decision-making process.

## Introduction

Colorectal cancer (CRC) is the third most commonly diagnosed malignancy and the second leading cause of cancer-related mortality worldwide, significantly impacting human life and health [1]. Despite advancements in medical technology benefiting CRC patients, the disease still exhibits high recurrence and metastasis rates [2]. Notably, there is a rising incidence of early-onset CRC (before age 50 years) [3, 4], indicating that patients are becoming younger. Therefore, identifying more precise predictive markers holds great significance for improving CRC treatment and prognosis.

Inflammation plays an important role in the pathogenesis and progression of CRC [5]. Accumulating evidence indicates that inflammatory markers, such as the lymphocyte-to-monocyte ratio, neutrophil-to-lymphocyte ratio, and systemic immune-inflammatory response index, serve as robust prognostic markers for CRC patients [6–8]. The pan-immune-inflammation value (PIV), a novel immune-inflammation marker, encompasses neutrophils, lymphocytes, platelets, and monocytes, offering a comprehensive assessment of cancer prognosis [9]. It has been validated as a potent predictor for the treatment outcomes of advanced cancers [10, 11]. Nonetheless, investigations into PIV’s correlation with CRC prognosis remain limited.

Dysregulated cholesterol metabolism is a prominent focus in the study of malignant tumor pathogenesis, with the mevalonate pathway within this process confirmed to be associated with the regulation of various cancers [12]. Research has shown that sustained cholesterol accumulation is a crucial prerequisite for CRC cells to acquire proliferative abilities, and it correlates with the activation or inhibition of multiple targets within metabolic pathways [13, 14]. High-density lipoprotein (HDL), as a constituent lipoprotein component, facilitates the reverse transport of cholesterol from peripheral tissues to the liver for catabolism. It serves as the primary substance in blood that promotes cholesterol metabolism and reduces its levels [15]. Beyond its lipid-transport roles, HDL possesses multifaceted functions including antioxidant, anti-inflammation, and immunomodulatory [16, 17]. The monocyte to HDL ratio (MHR), a validated inflammatory marker extensively utilized in cardiovascular diseases, has also been applied across various diseases [18–20]. It can be used as a reliable clinical indicator to assess the body’s cholesterol metabolism. Recent findings suggest that an increasing MHR is an independent risk factor for CRC [21].However, its potential value in oncology remains largely unexplored.

The purpose of this study is to use the preoperative composite inflammatory marker PIV and the lipid inflammatory marker MHR to reflect inflammation, immunity, and cholesterol metabolism, By integrating these aspects, we aim to explore their relationship with the prognosis of CRC and to develop a novel nomogram model for predicting the survival outcomes of CRC patients. This nomogram will offer a more comprehensive and systematic reference for prognostic evaluation in CRC patients, thereby providing valuable support and guidance for clinical decision-making.

## Materials and methods

### Study patients

A total of 172 patients with colorectal cancer who underwent radical resection at the Second Hospital of Lanzhou University between March 2018 and May 2019 were enrolled in this study. The inclusion criteria were as follows: (1) underwent radical resection; (2) pathologically diagnosed with CRC; (3) complete clinicopathological and follow-up data. Exclusion criteria comprised: (1) concurrent presence of other malignant tumors; (2) previous history of hematological diseases, autoimmune diseases, or chronic inflammatory diseases.

This study was conducted in accordance with the Declaration of Helsinki and approved by the Medical Ethics Committee of the Second Hospital of Lanzhou University (Project Number:2024 A-369). As this study is a retrospective and the privacy and personal identity information of the patients were protected, the need for informed consent was waived by the Medical Ethics Committee of the Second Hospital of Lanzhou University.

### Follow-up

The primary endpoint of the study was overall survival (OS). Survival time was defined as the duration from surgery to all-cause mortality or the last follow-up. Follow-up was diligently conducted for all participants, extending until either the endpoint of death or June 2022.

### Data collection

The data of all patients were obtained from the electronic medical record of the hospital information system. The collected data include a comprehensive array of variables: demographic details (sex and age), preoperative laboratory findings (blood cell counts, carcinoembryonic antigen (CEA), and HDL), and detailed pathological information. The latter included T stage, lymph node metastasis, overall TNM stage (tumors staged according to the eighth version of the American Joint Committee on Cancer (AJCC) tumor-node-metastasis classification), tumor location, grade, size, and the presence of neural or vascular invasion.

PIV= (neutrophil count × monocyte count × platelet count)/lymphocyte count [9], MHR = monocytes count /HDL [18].

### Statistical analysis

In this study, SPSS (version 26.0) and R software (version 4.3.2) were used for statistical analysis, and X-tile software (version 3.6.1) was used to calculate the optimal cut-off values of PIV and MHR. The Chi-square test or Fisher’s exact test was used to evaluate the relationship between preoperative PIV and MHR levels and clinicopathological characteristics of patients. Univariate and multivariate analyses were performed using Cox proportional hazards regression to screen the independent prognostic factors. The Kaplan-Meier method was used to generate the survival curve, and the parallel log-rank test was performed. A nomogram model predicting 1-, 2-, and 3-year OS after surgery was constructed based on the independent prognostic factors identified by multivariate Cox regression analysis. The C-index, receiver operating characteristic (ROC) curve, and area under curve (AUC) were used to evaluate the clinical prediction efficiency and discrimination of the nomogram. Calibration curves were employed to assess the consistency between predicted and observed survival. Decision curve analysis (DCA) was used to evaluate the clinical utility of the nomogram. A two-tailed *P* < 0.05 was considered statistically significant.

## Results

### Relationships between preoperative PIV and MHR levels and clinicopathological characteristics

In this study, 172 patients diagnosed with CRC were included. Using X-tile software, the optimal cut-off values for PIV and MHR were determined to be 265.75 and 0.26, respectively. Among these patients, the group with low preoperative PIV levels comprised 107 individuals (62.2%), whereas the group with high preoperative PIV levels contained for 65 individuals (37.8%). Additionally, the low preoperative MHR level group included 56 cases (32.6%), and the high preoperative MHR level group contained 116 cases (67.4%). Survival analysis revealed OS rates at 1-year, 2-years, and 3-years post- radical resection to be 91.8%, 76.1%, and 66.3%, respectively.

Table 1 summarizes the relationships between preoperative PIV and MHR levels and clinicopathological characteristics. The PIV was significantly associated with tumor location (*P* < 0.001), tumor maximum diameter (*P* = 0.008), and T stage (*P* = 0.019); however, no significant correlations were observed with other clinicopathological characteristics (all *P* > 0.05). The MHR was closely related to gender (*P* = 0.016), tumor maximum diameter (*P* = 0.002), and T stage (*P* = 0.038). Nevertheless, no significant correlations were found with other clinicopathological characteristics (all *P* > 0.05).

**Table 1:** Relationship between preoperative PIV and MHR levels and clinicopathologic characteristics of colorectal cancer patients

Variable	PIV					MHR			
	< 265.75	≥ 265.75	χ2	*P*		< 0.26	≥ 0.26	χ2	*P*
Age(years)			3.583	0.058				0.052	0.820
<60	45(42.1)	37(56.9)				26(46.4)	56(48.3)		
≥ 60	62(57.9)	28(43.1)				30(53.8)	60(51.7)		
Gender			0.838	0.360				5.814	**0.016**
Male	70(65.4)	38(58.5)				28(50.0)	80(69.0)		
Female	37(34.6)	27(41.5)				28(50.0)	36(31.0)		
CEA (µg/L)			0.687	0.407				1.677	0.195
Normal	49(45.8)	34(52.3)				31(55.4)	52(44.8)		
Elevated	58(54.2)	31(47.7)				25(44.6)	64(55.2)		
Tumor location			13.199	**< 0.001**				3.056	0.080
Colon	58(54.2)	53(81.5)				31(55.4)	80(69.0)		
Rectum	49(45.8)	12(18.5)				25(44.6)	36(31.0)		
Tumor maximum diameter(cm)			6.986	**0.008**				9.334	**0.002**
<5	65(60.7)	26(40.0)				39(69.6)	52(44.8)		
≥ 5	42(39.3)	39(60.0)				17(30.4)	64(55.2)		
T stage			5.467	**0.019**				4.315	**0.038**
T1-T2	25(23.4)	6(9.0)				15(26.8)	16(13.8)		
T3-T4	82(76.6)	59(91.0)				41(73.2)	100(86.2)		
Lymph node metastasis			1.897	0.168				1.258	0.262
No	56(52.3)	41(63.1)				35(62.5)	62(53.4)		
Yes	51(47.7)	24(36.9)				21(37.5)	54(46.6)		
TNM stage			0.612	0.434				1.232	0.267
I-II	56(52.3)	38(58.5)				34(60.7)	60(51.7)		
III-IV	51(47.7)	27(41.5)				22(39.3)	56(48.3)		
Grade			1.647	0.199				1.649	0.199
Well/Moderate	79(73.8)	42(64.6)				43(76.8)	78(67.2)		
Poor/Undifferentiated	28(26.2)	23(35.4)				13(23.2)	38(32.8)		
Neural or vascular invasion			0.407	0.523				3.101	0.078
No	38(35.5)	20(30.8)				24(42.9)	34(29.3)		
Yes	69(64.5)	45(69.2)				32(57.1)	82(70.7)		

### Survival analysis based on preoperative PIV and MHR

**Fig. 1:**
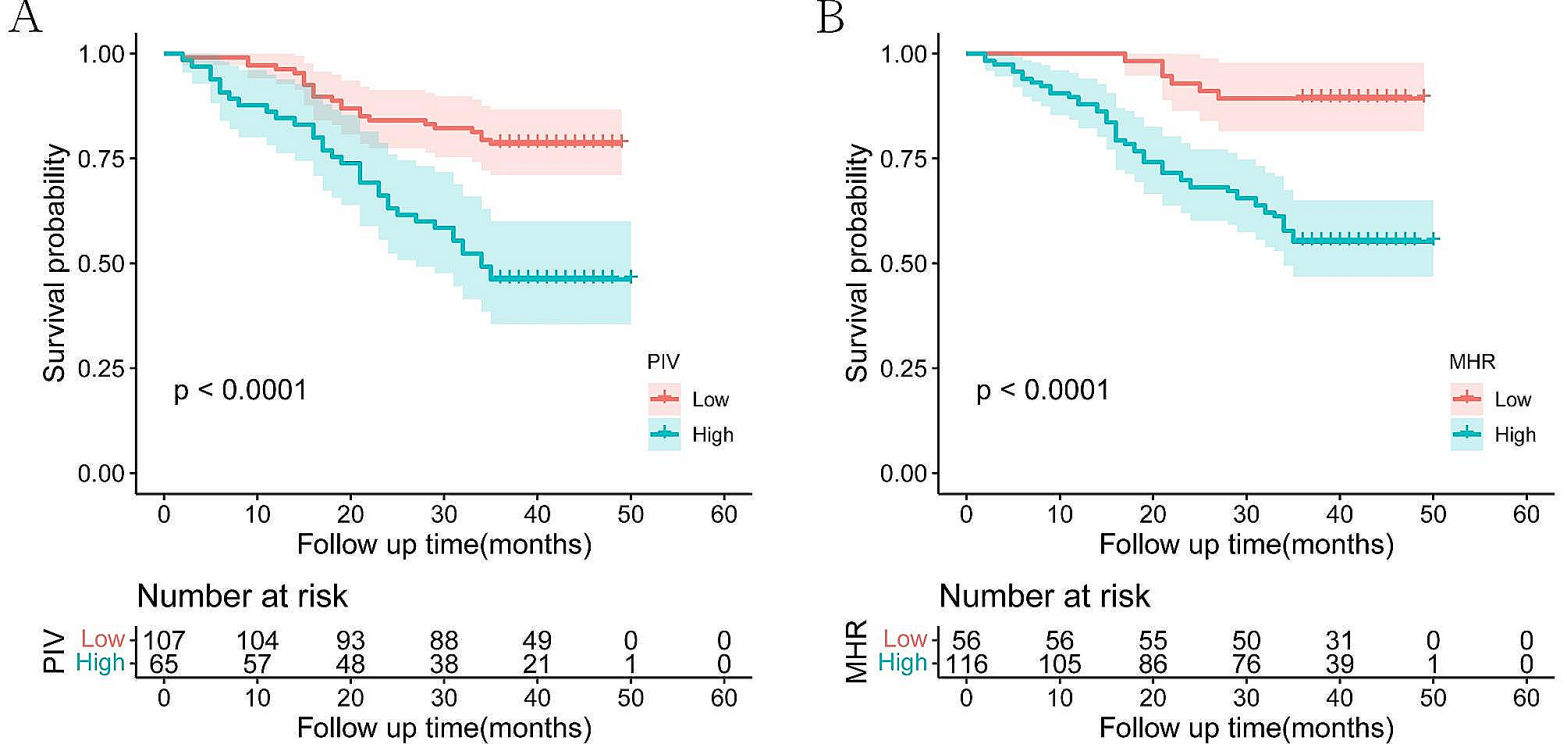
Kaplan–Meier curves of OS in patients with CRC by PIV(**A**), MHR(**B**)

The Kaplan-Meier survival analysis elucidated a significant disparity in 3-year OS rates between groups. Patients with high preoperative levels of PIV exhibited a markedly lower 3-year OS rates compared to those with low preoperative levels (46.1% vs. 78.5%, *P* < 0.0001; Fig. 1A). A parallel trend was observed in the context of MHR, where the groups with high preoperative MHR levels demonstrated significantly reduced 3-year OS rates relative to their counterparts with low preoperative levels (55.2% vs. 89.2%, *P* < 0.0001; Fig. 1B).

### Univariate and multivariate cox regression analyses

Table 2 presents the outcomes of both univariate and multivariate Cox regression analyses, elucidating the relationship of various clinical and biochemical parameters with OS in CRC patients. Univariate analysis pointed out that PIV, MHR, CEA, tumor location, grade and TNM stage were significantly correlated with OS (all *P* < 0.05). These variables were subsequently integrated into a multivariate regression framework to ascertain their independent prognostic value. Multivariate analysis results showed that PIV (Hazard Ratio (HR) = 2.476, 95% Confidence Interval (CI) = 1.410–4.348, *P* = 0.002), MHR (HR = 3.803, 95% CI = 1.609–8.989, *P* = 0.002), CEA (HR = 1.977, 95% CI = 1.121–3.485, *P* = 0.019), and TNM stage (HR = 1.759, 95% CI = 1.010–3.063, *P* = 0.046) emerged as independent prognostic indicators for OS.

**Table 2:** Univariate and multivariate analysis of poor prognostic factors in patients with colorectal cancer

Variable	Univariate analysis			Multivariate analysis	
	HR (95%CI)	*P*		HR (95%CI)	*P*
Age(years)					
<60	Reference				
≥ 60	1.347(0.801–2.265)	0.261			
Gender					
Male	Reference				
Female	1.126(0.665–1.906)	0.658			
CEA (µg/L)					
Normal	Reference			Reference	
Elevated	2.173(1.255–3.762)	**0.006**		1.977(1.121–3.485)	**0.019**
PIV					
<265.75	Reference			Reference	
≥ 265.75	3.075(1.815–5.210)	**< 0.001**		2.476(1.410–4.348)	**0.002**
MHR					
<0.26	Reference			Reference	
≥ 0.26	5.237(2.248–12.20)	**< 0.001**		3.803(1.609–8.989)	**0.002**
Tumor location					
Colon	Reference			Reference	
Rectum	0.474(0.256–0.879)	**0.018**		0.705(0.372–1.337)	0.285
Tumor maximum diameter(cm)					
<5	Reference				
≥ 5	1.105(0.660–1.849)	0.704			
T stage					
T1-T2	Reference				
T3-T4	2.037(0.875–4.744)	0.099			
Lymph node metastasis					
No	Reference				
Yes	1.670(0.997–2.799)	0.051			
TNM stage					
I-II	Reference			Reference	
III-IV	1.937(1.148–3.268)	**0.013**		1.759(1.010–3.063)	**0.046**
Grade					
Well/Moderate	Reference			Reference	
Poor/Undifferentiated	1.925(1.141–3.246)	**0.014**		1.375(0.795–2.379)	0.254
Neural or vascular invasion					
No	Reference				
Yes	1.415(0.795–2.516)	0.238			

### Development and validation of the nomogram model

**Fig. 2:**
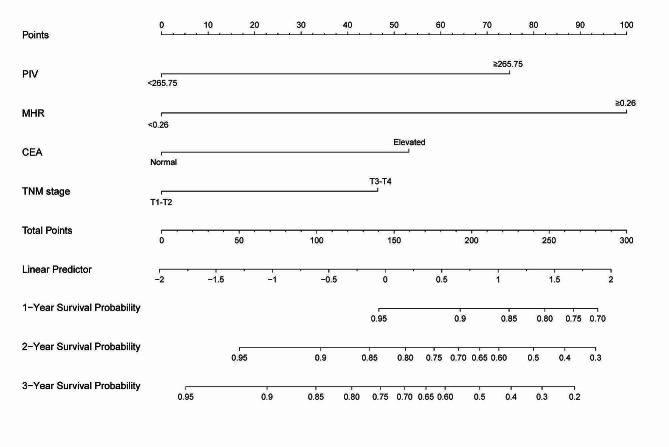
Nomogram model predicting 1-, 2- and 3-year OS in patients with CRC

**Fig. 3:**
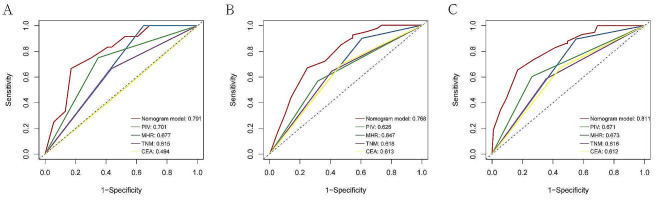
ROC curve of the nomogram predicting 1-year(**A**), 2- year(**B**), 3-year (**C**) survival in patients with CRC

**Fig. 4:**
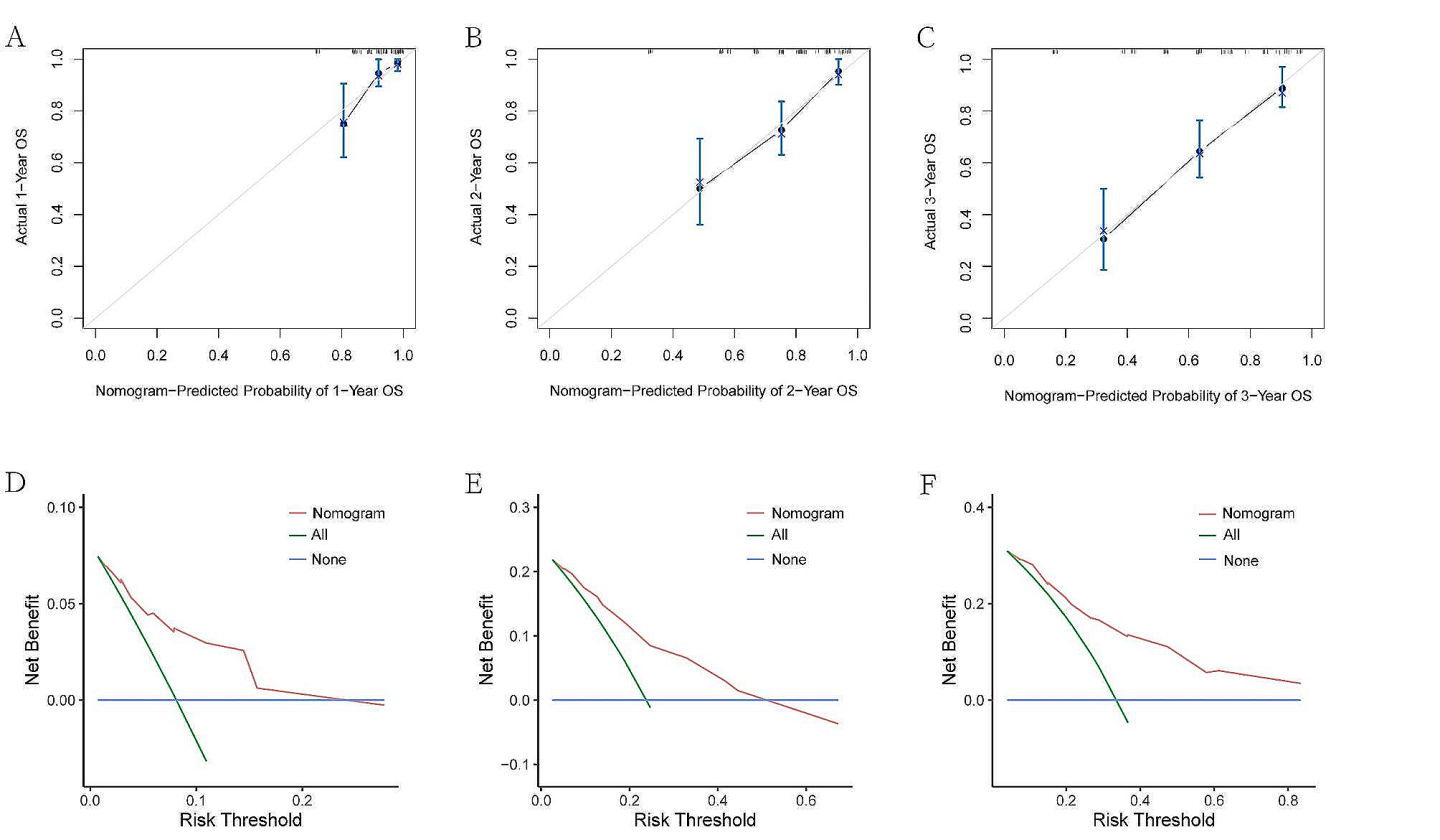
The calibration curves of the nomogram for predicting 1-year(**A**), 2- year(**B**), 3-year (**C**) OS probability in patients with CRC. Decision curve analyses with clinical net benefits of the nomogram at 1-year(**D**), 2-year(**E**)and 3-year(**F**)

Utilizing independent prognostic factors identified through univariate and multivariate Cox regression analyses, a nomogram model was constructed to predict 1-year, 2-year, and 3-year OS in CRC patients (Fig. 2). The predictive ability of the model was demonstrated by the consistency index (C-index). Through bootstrap validation (resampling = 1000), the C-index value was 0.748 (95% CI = 0.688–0.794), indicating substantial predictive accuracy. Comparative analysis between each independent risk factor and the nomogram model demonstrated superior predictive efficacy of the latter in predicting OS at 1-, 2-, and 3-year. The ROC curve results showed that the AUC values for 1-, 2-, and 3-year are 0.791,0.768,0.811, respectively (Fig. 3A-C). Calibration curves for 1-, 2-, and 3-year survival probabilities demonstrated a high level of agreement between predicted and observed outcomes, attesting to the model’s credibility (Fig. 4A-C). Additionally, the DCA further confirmed the significant clinical efficacy of this nomogram model in predicting survival probabilities at 1-, 2-, and 3-year (Fig. 4D-F).

### Risk stratification of the nomogram

**Fig. 5:**
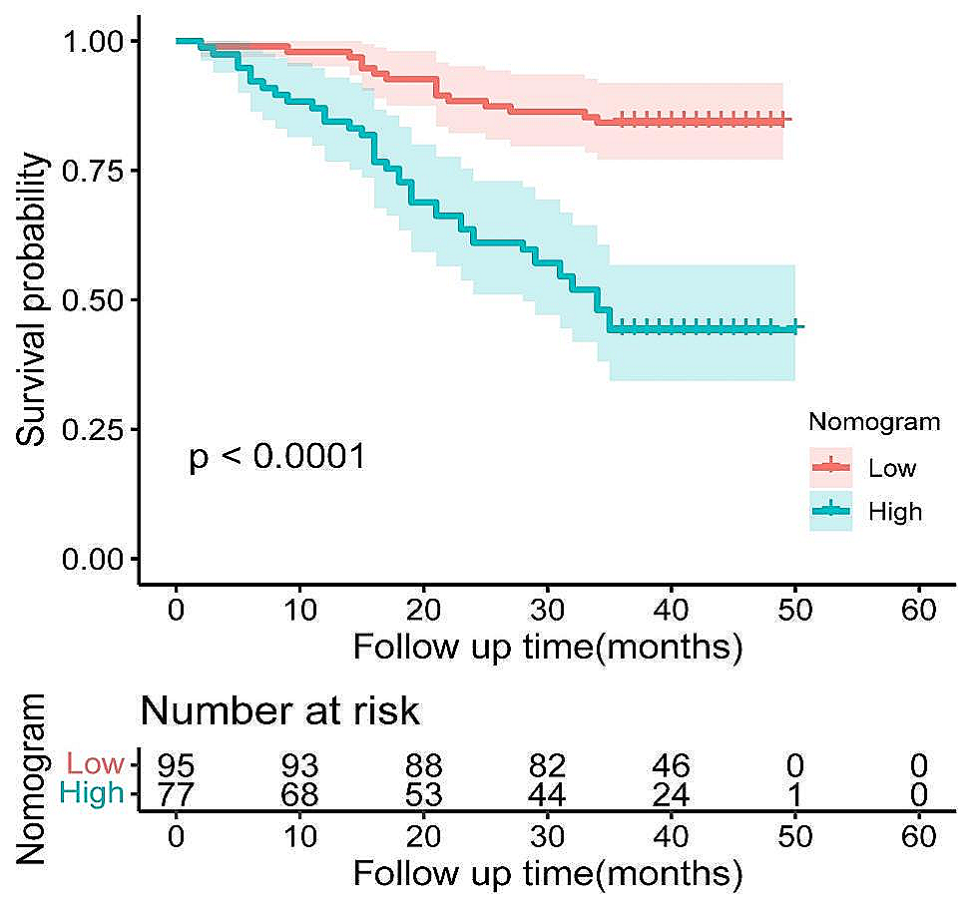
Kaplan-Meier survival curves of CRC patients with different risks stratified by the nomogram

Total points were calculated according to the nomogram model, and the X-tile software was used to ascertain an optimal cut-off value of 174.8. Subsequently, patients were accordingly categorized into two distinct groups: a low-risk group, characterized by a total nomogram score of less than 174.8, and a high-risk group, with scores equal to or exceeding 174.8. Kaplan-Meier survival analysis was then conducted to evaluate the differential survival outcomes between these groups. The analysis revealed a marked disparity in survival prognosis, with the low-risk group exhibiting significantly better survival outcomes compared to the high-risk group (*P* < 0.0001; Fig. 5).

## Discussion

As a malignant neoplasm of the gastrointestinal tract associated with increasing morbidity and mortality rates, the prognosis of CRC patients remains a significant challenge for medical professionals and researchers. Inflammatory, immune, and cholesterol metabolism factors have progressively gained attention in the pathogenesis and progression of CRC. Therefore, it is imperative to integrate these three factors to investigate their correlation with the prognosis of CRC, laying a foundation for early identification of high-risk patients and personalized treatment.

Inflammation is intricately associated with cancer, facilitating its development and promoting all stages of tumor formation [22]. Neutrophils exhibit dual roles in tumor biology, as they can eliminate tumor cells under certain conditions while also directly fostering tumor progression, metastasis, and angiogenesis [23]. Lymphocytes, serving as the primary constituents of the tumor immune barrier, initiate immune responses and execute direct killing of tumor cells by recognizing specific antigens [24]. Platelets contribute to tumorigenesis and angiogenesis through the secretion of mediators like platelet endothelial growth factor and platelet-derived growth factor; additionally, they aid cancer cells in evading immune surveillance [25, 26]. Monocytes exert diverse effects on the tumor microenvironment by inducing immune tolerance and enhancing the dissemination of tumor cells, and can also generate anti-tumor responses and activate antigen-presenting cells to elicit anti-tumor effects [27]. PIV encompasses these four cell types comprehensively reflecting both inflammatory response dynamics and immune status within an organism. Our study has shown that CRC patients with low preoperative PIV levels exhibit a more favorable prognosis compared to those with high preoperative PIV levels, indicating that PIV is a powerful indicator for predicting the prognosis of CRC patients, which is consistent with previous research conducted by Fuca et al. [9] and Corti et al. [28].

Dysregulated cholesterol metabolism constitutes another pivotal factor implicated in the pathogenesis and progression of CRC, a malignancy closely associated with dietary habits. Results from a comprehensive investigation into dietary patterns and incidence rates among Asian populations have indicated a significant escalation in CRC risk correlated with increased consumption of high-cholesterol foods [29]. Additionally, a meta-analysis has highlighted that statins not only have the potential to reduce CRC risk but also improve survival by counteracting cholesterol accumulation [30]. HDL, the sole lipoprotein capable of reverse cholesterol transport in plasma, has garnered increasing attention for its role in cancer prognostication, exerting anti-tumor effects through cholesterol reduction. Su et al. [31], utilizing mouse models demonstrated that simulating a high-HDL environment with HDL-like substances significantly inhibited lipid product accumulation in murine colonic adenomas, reduced tumor burden, and improved long-term survival. Studies have shown that patients with stage II/III CRC exhibiting elevated levels of HDL had better a prognosis compared to those with low HDL levels, evidenced by a 5-year OS rate of 85.3% (*P* = 0.002) [32]. Furthermore, HDL influences monocyte development, negatively regulates monocyte proliferation, suppresses the expression of endothelial cell adhesion molecules, and reduces monocyte migration to inflammatory sites [33, 34]. MHR, as an integrated marker combining inflammation and cholesterol metabolism, has demonstrated prognostic value across various diseases [18, 21]. A retrospective study identified high MHR levels as an unfavorable prognostic factor for patients who had undergone radical gastrectomy [35]. Previous studies have shown that HDL levels are higher in women than in men [36], potentially contributing to lower MHR levels in female patients. Interestingly, female CRC patients exhibited a more favorable prognosis than their male counterparts [1], suggesting a possible association between MHR levels and CRC prognosis. This association was confirmed in our study, our study found that preoperative high MHR levels were associated with poor prognosis in CRC patients.

Through prognostic analysis of 172 CRC patients who underwent radical surgery, we observed that high preoperative levels of the PIV and the MHR were associated with a poorer prognosis. PIV and MHR emerged as independent risk factors for OS, reflecting the dual impact of inflammation and cholesterol metabolism on the prognosis of CRC patients, and they can effectively predict outcomes. We constructed a novel nomogram model to predict 1-year, 2-year, and 3-year survival outcomes for CRC patients. The ROC curve analysis indicated superior predictive efficacy of the nomogram model compared to individual indicators alone. The nomogram provides a more nuanced risk stratification by combining inflammation and cholesterol metabolism indicators with traditional factors. This allows for a more precise assessment of a patient’s prognosis, facilitating tailored treatment approaches based on individual risk profiles. Clinicians can use the nomogram to identify high-risk patients who may benefit from more aggressive treatment modalities, closer monitoring and novel combinations of drugs targeting specific inflammatory pathways or cholesterol metabolism targets, thereby improving treatment outcomes. Fluctuations in PIV or MHR levels during treatment may also alert clinicians to reevaluate current treatment methods and make necessary adjustments. Patients with persistently high levels of PIV and MHR may require more intensive follow-up and earlier interventions, including lifestyle and clinical measures that can modify risk factors related to inflammation and cholesterol metabolism. Therefore, PIV and MHR levels in patients with CRC should be closely monitored during treatment.

To our knowledge, this study is the first to use inflammatory markers to reflect inflammation, immunity, and cholesterol metabolism, combining these aspects to explore their relationship with the prognosis of CRC patients. A nomogram model was constructed to visualize the impact of inflammation, immunity, and cholesterol metabolism on the survival outcomes of CRC patients. This nomogram allows for the rapid determination of patient risk levels through simple interpretation of test indices or results, allowing the more accurate and reliable identification of high-risk patients. By providing a holistic assessment of each patient’s unique clinical context, the nomogram serves as an invaluable resource for informing therapeutic strategies. Our nomogram offers clinicians a robust basis for making informed decisions, significantly enriching the potential for tailored patient management, holding substantial clinical importance.

However, our study also has some limitations. Firstly, it lacks external validation as it is a single-center retrospective study with a small sample size and limited geographical scope. Moreover, the follow-up period for participants was relatively brief, precluding the availability of long-term survival data beyond five years for comprehensive validation. Consequently, there is a pressing need for future research to undertake multicenter, large-scale prospective studies accompanied by external validation to substantially bolster the reliability and scientific rigor of the outcomes.

## Conclusion

In conclusion, preoperative PIV and MHR are independent risk factors for CRC prognosis. By integrating markers that reflect inflammation, immunity, and cholesterol metabolism, we have developed a novel nomogram model. This model serves as a robust tool for identifying patients at high risk of adverse outcomes, thereby enhancing the clinical decision-making process. Its application has the potential to influence patient management strategies, tailoring interventions to individual risk profiles and improving prognosis in CRC patients.

## Data Availability

The datasets generated and analysed during the current study are available from the corresponding author on reasonable request.
